# Engineering a high-affinity humanized anti-CD24 antibody to target hepatocellular carcinoma by a novel CDR grafting design

**DOI:** 10.18632/oncotarget.17228

**Published:** 2017-04-19

**Authors:** Fumou Sun, Tong Wang, Jiahao Jiang, Yang Wang, Zhaoxiong Ma, Zhaoting Li, Yue Han, Mingzhu Pan, Jialing Cai, Min Wang, Juan Zhang

**Affiliations:** ^1^ Antibody Engineering Laboratory, State Key Laboratory of Natural Medicines, Department of Molecular Biology, School of Life Science & Technology, China Pharmaceutical University, Nanjing 210009, PR China

**Keywords:** cluster of differentiation 24 (CD24), humanization, molecular operating environment (MOE), complementarity determining region (CDR) grafting, canonical residues

## Abstract

Cluster of differentiation 24 (CD24) is a specific surface marker involved in the tumorigenesis and progression of hepatocellular carcinoma (HCC). However, all reported anti-CD24 antibodies are murine ones with inevitable immunogenicity. To address this, a method using both molecular structure and docking-based complementarity determining region (CDR) grafting was employed for humanization. After xenogeneic CDR grafting into a human antibody, three types of canonical residues (in the VL/VH interface core, in the loop foundation, and interaction with loop residues) that support loop conformation and residues involved in the antigen-binding interface were back-mutated. Four engineered antibodies were produced, among which hG7-BM3 has virtually identical 3-D structure and affinity parameters with the parental chimeric antibody cG7. *In vitro*, hG7-BM3 demonstrated superior immunogenicity and serum stability to cG7. Antibody-dependent cellular cytotoxicity (ADCC), tumor cell internalization and *in vivo* targeting assays indicate that hG7-BM3 has the potential for development as an antibody-drug conjugate (ADC). We therefore generated the hG7-BM3-VcMMAE conjugate, which was shown to induce tumor cell apoptosis and effectively suppress nude mice bearing HCC xenografts. In conclusion, our study provides new inspiration for antibody humanization and an ADC candidate for laboratory study and clinical applications.

## INTRODUCTION

According to global cancer statistics, hepatocellular carcinoma (HCC) is the second leading cause of cancer mortality in developing countries and the sixth leading cause among males in developed countries [1]. Global precision medicine strategies call for new biomarkers in targeted therapies. However, few treatable molecular targets in HCC have been defined [2, 3]. Cluster of differentiation 24 (CD24) is a glycosylphosphatidylinositol (GPI)-linked membrane protein with high glycosylation activity and is localized in lipid membrane raft domains [4–7]. It is also a receptor that mediates antibody internalization [8, 9]. In particular, CD24 is known as a functional liver tumor-initiating cell (T-IC) marker and is upregulated in chemoresistant residual liver tumors [10]. Previously, we developed an anti-CD24 antibody series that targeted HCC both *in vitro* and *in vivo* [11, 12]. However, the immunogenicity of these murine-based antibodies will be an obstacle in future clinical use, especially in oncotherapy, where large doses and repeated administration are necessary to achieve significant efficacy [13, 14].

To reduce the immunogenic potential of murine antibodies while retaining full biological function, major efforts have been made [15, 16]. The generation of chimeric antibodies that graft murine variable domains onto human constant regions was the first step to reduce immunogenicity [17, 18]. Although the chimeric antibodies retained the parent antibody specificity and reduced immunogenicity substantially, their variable domains are still murine and have the potential to induce the human anti-mouse antibody (HAMA) response [19]. Therefore, recent studies have focused on developing humanized forms that can improve the potency of antibody-based treatment approaches. Grafting the complementarity-determining region (CDR) into a suitable human template is a widely-used method to humanize antibodies and can further reduce the HAMA response [20, 21]. Unfortunately, extensive sequence modifications within the framework regions (FR) may result in reduced or even lost binding affinities. Due to the FRs are missing the canonical residues that support CDR loop conformation and the residues involved in antigen contact [22, 23]. Some researchers suggested that these residues must be back-mutated to reconstitute full binding activity [24, 25]. However, how to identify these residues is unclear. These canonical residues often must be identified based on empirical knowledge rather than structural information, and interactional residues are often based on X-ray crystallization methods [26, 27]. These methods are cumbersome and lack rational guidance.

We previously generated a chimeric antibody cG7 specific for CD24. In this study, we identified the canonical residues based on a precise modeling and found interactional residues based on accurate molecular docking. Then, we back-mutated these residues following CDR grafting. After screening, hG7-BM3 was selected for its high binding affinity and reduced immunogenicity *in vitro* and specific targeting *in vivo*. Comprehensive evaluation showed that hG7-BM3 has the potential for further development as an ADC. Finally, we generated a hG7-BM3-VcMMAE conjugate that induced tumor cell apoptosis and showed superior anti-tumor activity *in vivo*.

## RESULTS

### Antibody modeling and evaluation

**Figure 1:**
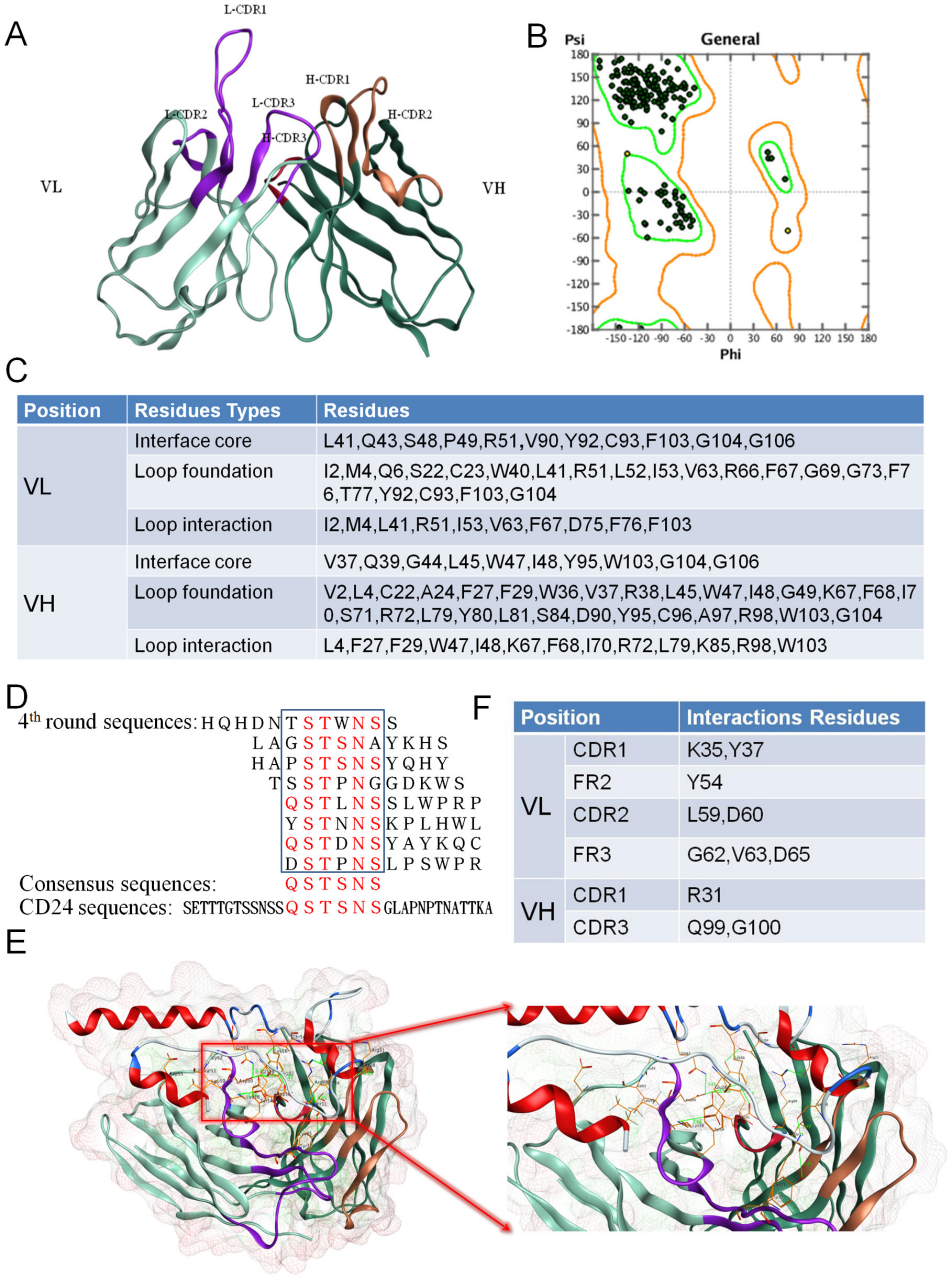
Identify the canonical residues supported CDR loop conformation and residues involved in contact with the antigen **(A)** 3-D structure of G7mAb Fv. MOE was be used to build the 3-D structure model of G7mAb Fv based on various antibody structures templates. **(B)** Fv structure evaluation with Ramachandran plot. Residues are all in the rendered regions. **(C)** Canonical residues in FRs. According to the 3-D structure, we identified three types of the canonical residues which were important for CDR conformation maintaining. **(D)** Epitope mapping of cG7. A dodecapeptide phage display library was screened against cG7. The consensus residues between the positive clones were QSTSNS, which were found in CD24 (residues 13-18). **(E)** Molecular docking of G7mAb Fv and CD24 based on epitope mapping. **(F)** Interactive residues in Fv. According to the complex structure, we identified the residues involved in contact with the antigen.

The amino acid sequences of G7mAb Fv were loaded into the MOE, and FRs or CDRs were identified by the Kabat numbering scheme. We selected the best scoring FRs template from PDB: 3SGD. Based on the loop length and similarity, the CDR loop templates were further assigned for CDR grafting. In loop grafting, redundant residues were deleted and the CDR loop templates were bonded to the FRs template. To relieve strained geometry and atom clashing, four rounds of tethered energy minimization were executed. We built the precise structure of G7mAb Fv using the MOE Antibody Builder module (Figure 1A). We used the geometry module to evaluate the stereo-chemical quality of the Fv structure. From the Ramachandran plot (Figure 1B), we determined that all residues were distributed in the allowed regions. (The green dots indicate the residues were in the core region, yellow indicates they were in the allowed region and red indicates they were in the outlier region.) Three types of canonical residues in FRs were identified by precise structural simulation of G7mAb Fv (Figure 1C). These canonical residues were recommended for humanization retention.

### Epitope mapping and molecular docking

The epitope of cG7 was mapped by screening a dodecapeptide phage display library. After four cycles of panning and enriching experiments, we sequenced the DNA sequences of phage clones, which gave an ELISA signal 3-fold greater than that of the control. As shown in Figure 1D, the consensus residues between positive clones were QSTSNS, which were found in CD24 (residues 13-18). ZDOCK was used to dock cG7 Fv and CD24. The superior modeled prediction and the residue binding sites are shown in Figure 1E. Through MOE analysis we know the interactions including hydrogen bonds, ion bonds or hydrophobic interactions, and involving the CD24 and cG7 Fv residues shown in Figure 1F. These residues play an important role in molecular interactions. The interactive cG7 Fv residues were mostly located in CDRs, and residues located in FRs were considered to be retained from the murine antibody during CDR grafting.

### Humanization of G7mAb Fv

**Figure 2:**
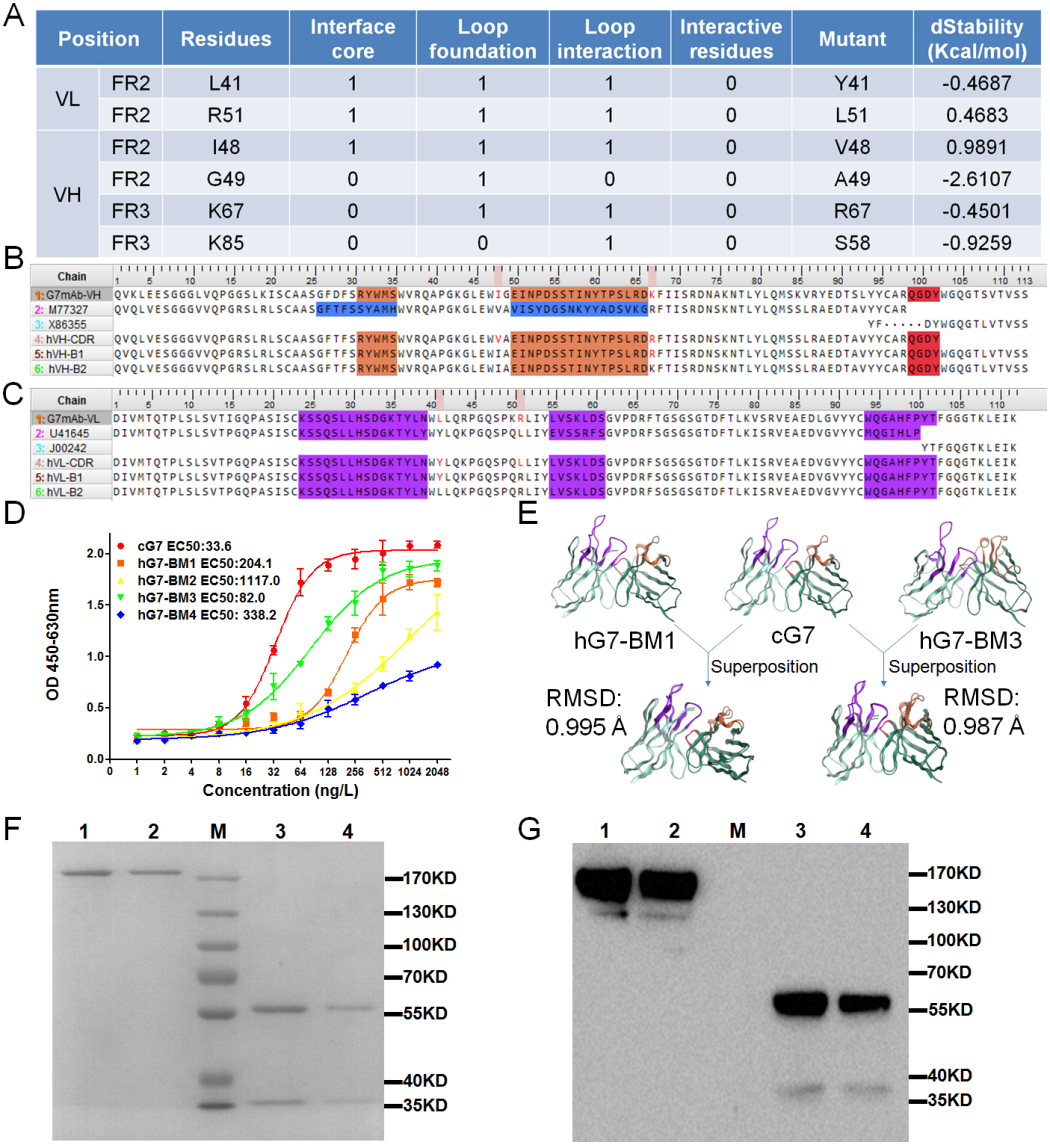
Design and structure prediction of humanized antibodies **(A)** The canonical and interactive residues different from the grafting Fvs. (1 represents yes, 0 represents no) And the calculation of antibody structure stability change upon mutation. **(B and C)** VH and VL sequence alignment. G7mAb-VH and G7mAb-VL were named for VH and VL regions of G7mAb murine antibody. M77327/X86355 found in V and J genes were chosen as human FRs donors for the humanized VH and U41645/J00242 for VL. **(D)** Antigen binding capacity of back mutate antibodies analyzed by ELISA. Binding curves of cG7 and hG7-BMs. **(E)** Superposed structure of G7mAb Fv and hG7-BM1/hG7-BM3. The calculated structural RMSD was 0.995 Å and 0.987 Å, respectively. **(F and G)** SDS-PAGE and Western Blot assay of stable expressed and purified hG7-BM1/hG7-BM3. Lane 1 and 2 were nonreduced hG7-BM1/hG7-BM3. Lane 3 and 4 were reduced hG7-BM1/hG7-BM3. Lane M were marker.

**Figure 3:**
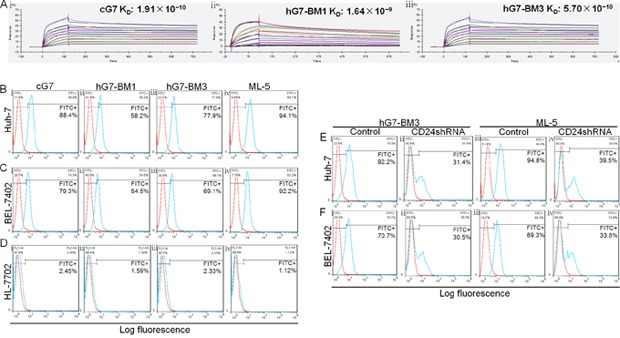
Binding affinity and binding capacity of humanized antibodies **(A)** Affinity of cG7, hG7-BM1 and hG7-BM3 to CD24-GST analyzed by SPR. *k_a_* and *k_d_* of hG7-BM3 **(Aiii)** were 7.99×10^5^ 1/Ms and 4.55×10^−4^ 1/s, and K_D_ was 5.70×10^−10^ M. cG7 **(Ai)**: *k_a_* was 1.76×10^6^1/Ms, *k_d_* was 3.36×10^−4^1/s, K_D_ was 1.91×10^−10^ M. hG7-BM1 **(Aii)**: *k_a_* was 4.65×10^5^1/Ms, *k_d_* was 7.64×10^−4^1/s, K_D_ was 1.64×10^−10^ M. **(B to F)** Binding capacity of humanized antibodies to hepatoma cell lines. **(Bi and Ci)** cG7 exhibited considerable affinity with two tumor cells (Huh-7 for 88.4%, BEL-7402 for 79.3%). **(Bii and Cii)** Compared with cG7, hG7-BM1 showed lower binding rate with Huh-7 (58.2%) and BEL-7402 (54.5%). **(Biii and Ciii)** hG7-BM3 showed similar binding capacity to cG7 (Huh-7 for 77.9%, BEL-7402 for 69.1%). **(Biv and Civ)** Two hepatoma cell lines showed high expression levels of CD24 (94.1% in Huh-7 and 92.2% in BEL-7402). **(Di to iv)** These antibodies showed no binding affinity with normal human hepatic cell line HL-7702. **(Ei, Eii and Fi, Fii)** When the CD24 knockdown, the binding rates of hG7-BM3 to Huh-7 and BEL-7402 were reduced to 31.4% and 30.5%. **(Eiii, Eiv and Fiii, Fiv)** The efficiency of CD24 knockdown in Huh-7 and BEL-7402 was 55.3% and 55.7%.

Human immunoglobulin germline V and J genes for FR donors were searched based on sequence identity in MOE. FR1-3 of the G7mAb VH region was selected to have the highest homology with the human germline V gene M77327|IGHV3-30*15, and FR4 was selected to have the highest homology with the J gene X86355|IGHJ4*02 (Figure 3A). FR1-3 of the VL region was selected to have homology with U41645|IGKV2-29*02, and FR4 was selected to have homology with the J gene J00242|IGKJ2*01. Then, we grafted CDRs onto human FRs and obtained the CDR grafting Fv named hVH-CDR and hVL-CDR. Next, we compared the CDR grafted Fv and G7mAb Fv. Eighteen residues in VH and nine residues in VL were different between the two sequences. Of these different residues, 4 residues in VH and 2 residues in VL belonged to canonical residues and none belonged to interactional residues. To maintain maximum affinity and reduce immunogenicity, we needed mutant minimum canonical residues. The residues scan module in MOE was used to analyze the dStability of the canonical residues selected to mutate. When humanized residues are mutated to murine residues, the change in stability will be reported as dStability, which is equivalent to the Boltzman average of the relative stabilities. From the results (Figure 2A), we chose to mutate the residues with more positive dStability values (L41, R51 in VL and I48, R67 in VH), which are, as a result, more unstable than other residues [28, 29]. As shown in Figure 2B and 2C, we back-mutated different residues in VH and VL and built 4 types of genes. These genes composed 4 types of antibodies: hG7-BM1 (hVH-B1, hVL-B1); hG7-BM2 (hVH-B1, hVL-B2); hG7-BM3 (hVH-B2, hVL-B1); and hG7-BM4 (hVH-B2, hVL-B2).

### Expression and comparison of humanized antibodies

cDNA for hG7-BMs was inserted into the expression vectors pMH3 and pCApuro. The plasmids were transfected into CHO-s cells, and after two cycles of screening, we obtained stable clones with high expression levels of hG7-BMs. SDS-PAGE and Western blotting were used to analyze the antibodies after purifying (Figure 2F and 2G). The binding activities of these five antibodies were analyzed and compared by ELISA. As shown in Figure 2D, the ELISA indicated that hG7-BM1 and hG7-BM3 had higher binding activities than the other humanized antibodies. Then, we used a superposition module in MOE to compare antibody structures, and the conformation difference was reported as the Root Mean Square Deviation (RMSD) value. The structure of G7mAb Fv was superposed with the hG7-BM1 or hG7-BM3 Fv structure (Figure 2E). The RMSD value between the G7mAb Fv and hG7-BM1 Fv structures was 0.995 Å and between the G7mAb Fv and hG7-BM3 Fv structures was 0.987 Å. These results indicate that hG7-BM3 Fv had a similar conformation to the parent G7mAb Fv.

### Binding affinity and binding capacity of humanized antibodies

The binding affinity and kinetics of CD24 to immobilized hG7-BM1 or hG7-BM3 were evaluated, and the 1:1 binding model was used for recorded sensorgrams. hG7-BM3 exhibited high affinity to CD24 (*k_a_* (1/Ms): 7.99×10^5^, *k_d_* (1/s): 4.55×10^−4^, K_D_ (M): 5.70×10^−10^) (Figure 3Aiii), which is comparable to that of cG7 (*k_a_* (1/Ms): 1.76×10^6^, *k_d_* (1/s): 3.36×10^−4^, K_D_ (M): 1.91×10^−10^) (Figure 3Ai). The affinity constant between hG7-BM1 and CD24 (*k_a_*(1/Ms): 4.65×10^5^, *k_d_* (1/s): 7.64×10^−4^, K_D_ (M): 1.64×10^−9^) (Figure 3Aii) was lower than that of its parental antibody. The humanized antibody hG7-BM3 demonstrated specificity and affinity to CD24, confirming that hG7-BM3 retained binding capacity *in vitro*.

Huh-7 and BEL-7402 are human hepatoma cell lines that express a CD24 ligand. HL-7702 is a normal human hepatic cell line. Compared to HL-7702 cells, Huh-7 and BEL-7402 cells showed high binding affinity to hG7-BM3 (Figure 3Biii, 3Ciii, 3Diii). hG7-BM3 and cG7 had similar binding capacities to Huh-7 and BEL-7402, and the binding capacity of hG7-BM1 was lower than that of its parental antibody (Figure 3Bi and Bii, 3Ci and 3Cii). To determine whether hG7-BM3 was bound to hepatoma cells through CD24, we used RNA interference to knockdown CD24 in hepatoma cells. Through the reduction of binding capacity of positive control ML-5, we calculated that the knockdown efficiency of CD24 in Huh-7 and BEL-7402 cells was 55.3% and 55.7%, respectively (Figure 3Eiii and 3Eiv, 3Fiii and 3Fiv). The binding of hG7-BM3 to two cell lines was significantly decreased (Figure 3Ei and 3Eii, 3Fi and 3Fii). Above, hG7-BM3 specifically bound to membrane CD24 in hepatoma cell lines, and its binding capacity was comparable to that of cG7.

### hG7-BM3 enhanced PBMCs or NK92-FcR cell cytotoxicity

**Figure 4:**
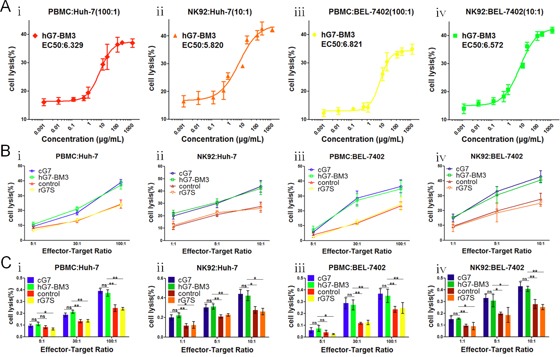
hG7-BM3 enhanced PBMCs or NK92-FcR cells cytotoxicity **(Ai to iv)** Cytotoxicity assay to assess the PBMC/NK92 cell-mediated killing of hepatoma cell cells. The EC_50_ of hG7-BM3 were fitted. **(Bi to iv)** Huh-7 and BEL-7402 were used as target cells and PBMC/NK92 cells as effect cells for a LDH release assay. The E:T ratios were indicated. The equivalent dose of rG7S with no Fc domain was used as scFv group. **(Ci to iv)** The increase of cell lysis was enhanced significantly upon treatment with hG7-BM3 compared to control groups or scFv group and unchanged upon treatment with hG7-BM3 compared to cG7. Data were given as the mean ± SD (n = 5). *p < 0.05, ***p < 0.001, ns: no significance.

To address whether hG7-BM3 still retains the ability of Fc-mediated ADCC, CD24^+^ HCC cells were chosen as target cells and PBMCs or NK92-FcR cells as effector cells. We first explored the cytotoxicity by serial concentrations of hG7-BM3 (from 0.001 μg/mL to 1000 μg/mL) at a fixed effector cell/target cell (E/T) ratio of 100:1 for PBMCs and 10:1 for NK92-FcR cells. Cells lysis was saturated with 100 μg/mL G7mAb in PBMCs and 100 μg/mL in NK92-FcR (Figure 4Ai to 4Aiv). Target cells were then co-cultured with PBMCs or NK92-FcR cells and subsequently treated with optimized concentrations of hG7-BM3. Cell lysis was similar in both cG7 and hG7-BM3, suggesting that hG7-BM3 retained the Fc-mediated ADCC effect for PBMCs with an E/T ratio of 100:1 (Figure 4Bi, 4Biii and 4Ci, 4Ciii). For the NK92-FcR group, 100 μg/mL of hG7-BM3 induced 42.2% lysis of Huh-7 cells and 40.7% of BEL-7402 cells at an E/T ratio of 10:1, which is close to the cG7-treated groups (Figure 4Bii, 4Biv and 4Cii, 4Civ). The overall data indicate that hG7-BM3 neither decreased its specificity nor avidity to CD24-positive cells and hG7-BM3 retained the Fc-mediated ADCC effect.

### Dynamics and targeting capability of hG7-BM3 *in vivo*

**Figure 5:**
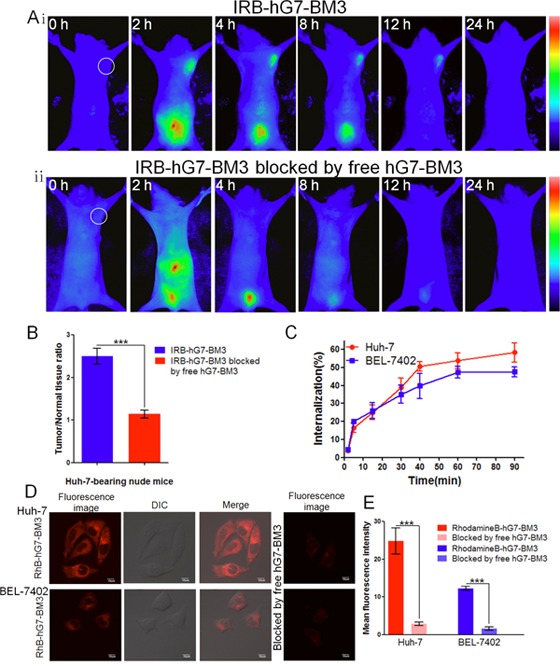
The tumor-targeting efficacy assay *in vivo* and tumor cell internalization assay *in vitro* **(Ai)** The bio-distribution of IRB-hG7-BM3 was evaluated by the NIR imaging assay in Huh-7-bearing nude mice within 24 h. **(Aii)** In blocking experiments, free hG7-BM3 inhibited the probes from binding to the tumor sites. **(B)** Tumor/normal tissue ratios calculated at 4-h post-injection of probe groups into Huh-7-bearing nude mice from the region of interest (ROIs). **(C)** hG7-BM3 internalized into hepatic cells rapidly within 40 min. The internalization rate was stabilizing at 90 min. **(D)** Laser confocal fluorescence microscopy images of Huh-7 and BEL-7402 cells incubated with the RhB-hG7-BM3 fluorescent probe, with or without a blocking dose of free hG7-BM3. **(E)** Mean fluorescence intensity of Huh-7 or BEL-7402 cells treated with RhB-hG7-BM3 probes, compared to blocking with free hG7-BM3. Data were given as the mean ± SD (n = 6). ***p < 0.001, ns: no significance.

After the injection of IRB-hG7-BM3, fluorescent signals spread throughout the body immediately. Four hours later, most fluorescent antibody was excreted through the kidneys, and Huh-7 xenografts were distinguished by fluorescence. The fluorescence signal was maintained for 12 h, and at 24 h a weak signal could still be detected (Figure 5Ai). In addition, the blocking group showed no intense fluorescent signals at the tumor site, indicating that tumor targeting is mediated by hG7-BM3 (Figure 5Aii). The fluorescent signals were significantly different, with a maximal tumor/normal tissue ratio (T/N ratio) at 4 h of 2.50 and 1.14 for the IRB-hG7-BM3-treated group and its blocking group, respectively (Figure 5B). These results indicate that hG7-BM3 effectively targeted the CD24^+^ HCC cells by specifically binding to CD24 *in vivo*.

### Cancer cell internalization

The CD24-mediated internalization rate of hG7-BM3 was quantified by flow cytometry and observed by confocal microscopy. As shown in Figure 5C, hG7-BM3 rapidly internalized into hepatic cells within 40 min. The internalization rate tended towards stability at 90 min and the rate of hG7-BM3 to Huh-7 cells was 58.3%, which was higher than hG7-BM3 to BEL-7402 cells (47.5%). From the confocal microscopy results, the fluorescence intensity of Huh-7 cells incubated with RhB-hG7-BM3 was higher than that of BEL-7402 cells (Figure 5D). These results are consistent with the flow cytometry results described above. In addition, Huh-7 and BEL-7402 exhibited a much higher mean fluorescence intensity (MFI) than their blocked counterparts (Figure 5E), indicating the specificity of hG7-BM3 to CD24 receptors.

### Generation and identification of hG7-BM3-VcMMAE

**Figure 6:**
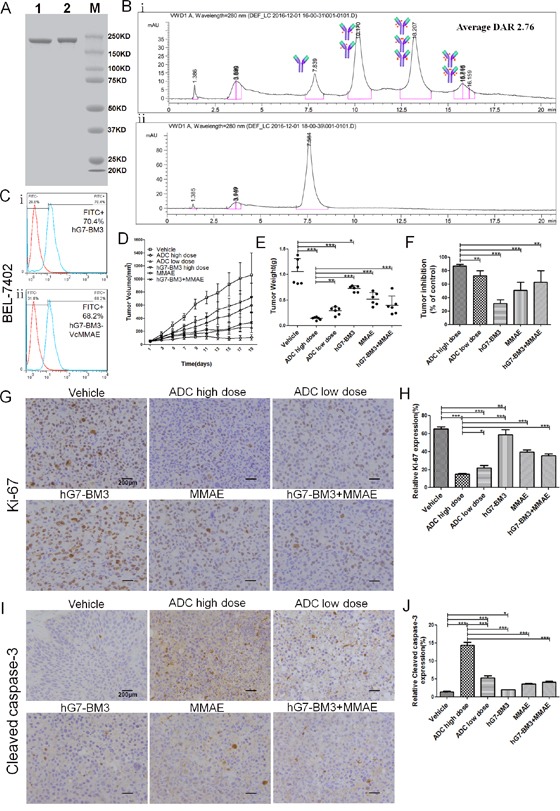
Characterization and *in vivo* anti-tumor activity of hG7-BM3-VcMMAE **(A)** Analysis of SDS-PAGE (8%) for purity, showing one major band of hG7-BM3-VcMMAE under non-reducing conditions. Lane 1 was hG7-BM3; Lane 2 was hG7-BM3-VcMMAE; and Lane M was the marker. **(B)** Hydrophobic interaction chromatography (HIC) analysis of hG7-BM3-VcMMAE on a butyl-NPR column yielded two predominant peaks corresponding to antibodies containing two and four drug molecules. **(C)** hG7-BM3-VcMMAE showed similar binding capacity to hG7-BM3. **(D)** Tumor growth curves of each group under different treatments. 1×10^7^ BEL-7402 cells were subcutaneously injected into 36 female BALB/c nude mice, which were then randomly divided into 6 groups (n = 6). When the average tumor volume reached 50 mm^3^, tumor-bearing mice were treated with hG7-BM3-VcMMAE and the tumor volume was measured. **(E)** The average tumor weights of different groups after 19 days of treatment. hG7-BM3-VcMMAE significantly improved the tumor inhibition rate compared to hG7-BM3 or MMAE. **(F)** Tumor inhibition rates of different groups. Data are given as the mean± SD (n= 6). **(G and I)** Expression levels of Ki67, cleaved caspase-3 (brown staining) in tumors analyzed by IHC. Photomicrographs were taken at 400× magnification. Scale bar = 200 μm. **(H and J)** The proliferation rate (Ki67) and the apoptosis rate (cleaved caspase-3) of the tumor cells were found in excised xenograft tumors and was defined as the percentage of stained cells. Data were given as the mean ± SD (n = 5). *p < 0.05, **p < 0.01, ***p < 0.001, ns: no significance.

hG7-BM3 was partially reduced to yield interchain free thiol groups and was then reacted with VcMMAE to produce hG7-BM3-VcMMAE. The identities of hG7-BM3-VcMMAE were confirmed by non-reducing SDS-PAGE (Figure 6A) and HIC (Figure 6B). hG7-BM3-VcMMAE migrated as a single major band with a molecular weight close to naked hG7-BM3. The molecular weight of VcMMAE was approximately 1.36 kDa, which was insignificant compared with 156 kDa in SDS-PAGE. Antibodies with different drug stoichiometries were identified by HIC. Four major peaks visible at 7.8, 10.2, 13.2 and 15.8 min suggest that hG7-BM3 was mainly conjugated with a two, four and six payload. The weighted average DAR was 2.76. As shown in Figure 6C, hG7-BM3-VcMMAE maintained a high binding capacity to BEL-7402 cells after revision.

### hG7-BM3-VcMMAE has anti-tumor efficacy in HCC-bearing mice

To evaluate the *in vivo* anti-tumor activity of hG7-BM3-VcMMAE, a BEL-7402 xenograft model was used. The growth of xenografted tumors was significantly inhibited in high dose hG7-BM3-VcMMAE-treated groups, while hG7-BM3 did not show an obvious anti-tumor effect (Figure 6D and 6E). Treatment with hG7-BM3-VcMMAE in tumor-bearing mice yielded maximal tumor inhibition rates of 86.7% and 72.5% at doses of 2 mg/kg and 0.5 mg/kg, respectively. As a reference for the humanized antibody, 2 mg/kg hG7-BM3 was not shown to achieve an obvious tumor inhibition rate (31.4%) with the same (P<0.001) (Figure 6F). The effect of hG7-BM3-VcMMAE on the mitotic index (Ki67) and apoptosis (cleaved caspase-3) in the tumor was detected by IHC staining. A distinct increase in cleaved caspase-3 and a reduction in Ki67 levels were observed after treatment with hG7-BM3-VcMMAE, which indicates that ADC inhibited tumor cell proliferation and induced apoptosis in tumor cells (Figure 6G, 6H,6I and 6J).

## DISCUSSION

The application of therapeutic antibodies for hepatocellular carcinoma is limited by the lack of specific antigens on tumor cells. CD24, a GPI-anchored protein that is overexpressed in multiple cancers, is a useful target antigen for HCC [10, 30, 31]. In previous study, we developed a CD24 targeted monoclonal antibody G7mAb based on hybridoma technology. Flow cytometry assays demonstrated specific binding of CD24^+^ Huh-7 and HT29 cells by G7mAb. And the near-infrared fluorescence imaging revealed that G7mAb aggregate in CD24^+^ Huh-7 hepatocellular carcinoma xenograft tissue via specific binding to CD24 *in vivo* [11]. In this study, we sought to humanize G7mAb because of its expected binding properties and potential broad clinical application. Various approaches have been developed to humanize murine mAbs. Among these methods, CDR-grafting remains a common and proven strategy for humanizing murine antibodies. Traditional CDR-grafting may result in a partial or complete loss of affinity of the grafted antibodies [22], and this can be remedied by back-mutating some residues of human origin to the corresponding murine ones. However, this high-throughput trial-and-error modality is tedious and involves expensive and time-consuming experiments. Back-mutation should be guided by identification of the decisive residues, which are the canonical residues that support the CDR loop conformation, and residues involved in contact with the antigen. Nevertheless, the canonical residues related to spatial conformation are often identified based on empirical knowledge, and the residues related to molecular interactions are often identified based on complex crystallization and X-ray crystallization methods. In this study, we devised a novel solution to the above problems: we found the canonical residues based on a modeling by MOE and identified interactional residues based on accurate molecular docking. Then, we back-mutated these residues following CDR grafting to reduce immunogenicity while retaining the maximum original binding affinity in G7mAb.

First, canonical residues related to spatial conformation were confirmed with structural information. A precise G7mAb Fv structure was modeled by MOE using antibody structure templates for all subdomains (Figure 1A). Canonical residues were identified and categorized into 3 types in light of the Fv structure (Figure 1C). These decisive residues were preserved from the murine antibody in CDR grafting. Second, residues involved in contact with the antigen were identified by accurate molecular docking. From a dodecapeptide phage display library, we screened the antigen epitope recognized by cG7. The molecular docking of cG7 Fv and epitope residues on CD24 by ZDOCK provided important residue interactions at the antibody-antigen binding site. These interactive residues were also retained in humanization. Though ZDOCK is a rigid protein docking program, we used only six epitope residues to establish the docking model. These greatly narrowed down the incorrect possibilities of docking. And this method is one of the potential alternatives to complicated X-ray crystallization method. Based on the above method, the canonical residues and interactive residues were identified accurately and rapidly.

After confirming the back-mutated residues, we humanized G7mAb Fv by CDR grafting. Human V or J genes with the highest sequence similarity were selected, and we conserved most canonical residues (Figure 2B). The humanized antibody hG7-BM3, which was back-mutated with the 3 residues, showed similar antigen binding capacities with the chimeric antibody cG7 (Figure 3). The 3-D structure of hG7-BM3 was superimposed with the G7mAb Fv structure, and the structural RMSD (0.987 Å) indicated a similar conformation between hG7-BM3 and the parental antibody. hG7-BM3 maintained similar binding affinity and binding activity to the parental chimeric antibody cG7. The high stability and reduced immunogenicity of hG7-BM3 *in vitro* suggests it is more stable and secure in the human body (Supplementary Figure 1 and Supplementary Figure 2). In ADCC tests, we found that hG7-BM3 can enhance PBMC and NK cell-mediated cell lysis of CD24^+^ HCC cells. Near-infrared fluorescence results showed that hG7-BM3 specifically targeted HCC xenografts, with a maximal tumor/normal tissue ratio of 2.50 for BEL-7402 cells at 4 h post-injection. Moreover, the binding of IRB-hG7-BM3 to antigens was blocked by free hG7-BM3. These results suggest that hG7-BM3 has high specificity and affinity to CD24^+^ HCC.

The development of antibody-drug conjugates (ADCs) through conjugating of cytotoxic agents to monoclonal antibodies has provided more tumour specificity and potency not achievable with traditional drugs. Several promising ADCs are now in late-phase clinical trials for the treatment of various human cancers [32]. And two ADCs have since achieved FDA approval: Trastuzumab emtansine (T-DM1) was approved for the treatment of metastatic breast cancer [33], and Brentuximab vedotin (SGN-35) was approved for the treatment of refractory Hodgkin's lymphoma and systemic anaplastic large-cell lymphoma [34]. CD24 is a receptor that mediates antibody internalization. For this reason, anti-CD24 antibodies have the potential to be developed as ADC drugs, and many researchers have attempted this strategy. For example, Barbara A. Froesch attempted to use the anti-CD24 antibody SWA11 to transport doxorubicin to human small cell lung cancer tumor lesions [8]. Encouragingly, our study suggests that the internalization rate of hG7-BM3 was desirable. Considering that the internalization rate of reported anti-CD24 antibodies was approximately 30% [8], our hG7-BM3 was internalized at a 58.3% rate to Huh-7 cells and a 47.5% rate to BEL-7402 cells. Target recognition, antibody affinity and internalization rate are important criteria for the development of effective ADC therapies. Fortunately, hG7-BM3 exhibited all of these properties. Therefore, we generated an hG7-BM3-VcMMAE conjugate, which increased the anti-tumor potency of hG7-BM3 and broadened the therapeutic window of MMAE [35, 36]. The observable decrease in tumor volume in the *in vivo* assay indicated that the anti-tumor efficacy of hG7-BM3-VcMMAE was dramatically improved in comparison with either parental hG7-BM3 or native MMAE alone. What's more, there was no weight loss observed in both groups treated with hG7-BM3-VcMMAE, indicating that the treatments were well tolerated (Supplementary Figure 3). In addition, the observed decrease in the expression of Ki67 and increase in cleaved caspase-3 (Figure 6G and 6I) indicates that hG7-BM3-VcMMAE-mediated tumor inhibition was associated with inhibition of tumor cell proliferation and the induction of cell apoptosis. Based on these exciting results, we will further develop the ADC for hG7-BM3. We will use site-specific drug conjugation to avoid differences in pharmacokinetics and pharmacodynamics and to ensure the safety of ADC drugs *in vivo* [37, 38].

In summary, we humanized G7mAb Fv by using a molecular structure and docking-based CDR-grafting method. It would be interesting to explore whether this efficient and reliable approach, which was developed using combined computational modeling and experimental methods, could be applicable to the humanization of other mouse monoclonal antibodies. Given the high target recognition, affinity and internalization rate, hG7-BM3 is a promising antibody for ADC development. As expected, hG7-BM3-VcMMAE exhibited ideal antitumor activity, and it is a potential anti-tumor drug candidate for clinical studies.

## MATERIALS AND METHODS

### Materials

Eukaryotic expression vectors pMH3, pCApuro contained full-length IgG1 H& L chain were preserved in our lab. Restriction enzymes, T4 DNA Ligase and PCR mastermix were purchased from Thermo Scientific (Shanghai, China). Chinese hamster ovary (CHO-s) cell line was purchased from AmProtein (Hangzhou, Zhejiang, China). Human hepatoma cell line Huh-7, BEL-7402 and human hepatic cell line HL-7702 were preserved in our lab. Cell culture media and trypsin powder were purchased from Life Technologies (Basel, Switzerland). Human peripheral blood (PBL) was obtained from the Blood Center of Jiangsu Province (Nanjing, China). Six-week-old female BALB/c nude mice were purchased from Comparative Medicine Centre of Yangzhou University (Yangzhou, China). All animals were treated following the standards of Comparative Medicine Centre of Yangzhou University and all animal experiments were conducted under protocols approved by the Animal of the Ministry of Health of the People's Republic of China (Document No. 55, 2001).

### Antibody modeling and evaluation

The amino acid sequence of murine antibody G7mAb Fv was determined in a previous study by our group. Molecular Operating Environment (MOE, version 2013.08) was used to build the 3-D structure model of G7mAb Fv, where we searched each subdomain structural template in the Protein Data Bank (PDB) database [39]. Sequence identity was used as the basis to search for templates of framework regions and sequence similarity was used to search for CDR regions. After choosing the best mode, we then refined the model to relieve strained geometry, further applied an energy minimization scheme in the Amber 10: EHT force field and achieved a final structure model. A protein geometry module was used to evaluate the structure model and draw a Ramachandran plot [40]. We identified three types of canonical residues in FRs that were important for maintenance of CDR conformation or for the binding affinity of the antibody. Type 1 consisted of the VH-VL interface core residues, which are major Fv dimer contact residues and play a critical part in the packing of two domains [41]. Type 2 consisted of the CDR loop foundation residues, which are buried in the VH/VL and are close to the CDR loop. Type 3 consisted of the CDR loop interaction residues, which contact directly with the CDR loop foundation residues through hydrogen and ionic bonds and by hydrophobicity. These 3 types of canonical residues were important for maintenance of CDR conformation and the binding affinity of the antibody.

### Epitope mapping and molecular docking

Ph.D.™-12 Phage Display Peptide Library Kit (New England Biolabs, Ipswich, MA, USA) was used to map epitopes on CD24 [42]. cG7 was coated in the plate for 12 h at 4°C and incubated with 1.5×10^10^ phages for 2 h at 37°C. After washing thrice with PBS, the bound phages were eluted and amplified in *E. coli* ER2738 for the next cycles. Four cycles later, high affinity individual clones were selected by ELISA and sequenced. Based on the sequencing results, we analyzed the cG7 epitope. The ZDOCK program (version 3.0.2) was used to dock cG7 and epitope residues on CD24 using a structure downloaded from the Protein Model Portal (PMP) [43, 44]. The superior modeled complex structure was analyzed by MOE and the residues that played an important role in the molecular interactions were identified.

### Humanization of G7mAb Fv

We searched the human immunoglobulin germline V genes and J genes database by the Fab-oriented sequence alignment method. The conserved template with the highest FR similarity and the most canonical residues was selected. Selected FRs were substituted for murine FRs, and CDR-grafted Fv (hVH-CDR, hVL-CDR) was archived. Then, the different residues, including the main chain and conserved residues, between the CDR-grafted Fv and G7mAb Fv were compared. We obtained a report for the different residues and evaluated the effect on antibody potency based on reports generated previously for the canonical residues. Last, we obtained the humanized Fv sequences based on CDR grafting and back-mutation.

### Construction, expression and purification of humanized antibodies

The cDNA sequences of humanized Fv were optimized to CHO-preferred codons and synthesized by GenScript (Nanjing, China). The VH DNA was digested with *EcoR*I and *Nhe*I and the VL DNA was digested with *EcoR*I and *Sal*I; the fragments were then ligated with digested pMH3-H, pCApuro-H, pMH3-L, and pCApuro-L, respectively [45]. The source of human constant region is from Cetuximab. Transfection, expression and purification were performed as described previously [45]. Humanized antibodies with high affinity were primarily screened by ELISA. The degree of similarity between cG7 Fv and humanized Fv was analyzed by the MOE superpose module, which also calculated the RMSD structure between corresponding protein alpha carbons (Cα) [46].

### Binding affinity and binding activity analysis

The binding affinity was assayed by ELISA and the Biacore X100 system (GE Healthcare, Buckinghamshire, UK). First, 1 μg/ml GST-CD24 was immobilized on 96-well plates at 4°C for 12 h. Then, a series of concentrations of purified cG7 or hG7s was incubated at 37°C for 1 h. To detect the HRP-conjugated goat-anti-human IgG H+L antibody, the plate was incubated at 37°C for 1 h. A BioTek Synergy 2 plate reader was used to monitor the difference in absorbance between 450 nm and 630 nm. The association rate constant *ka*, the dissociation rate constant *kd* and the equilibrium constant *KD* (*kd/ka*) was assayed by Biacore as described previously [45]. The binding activity was assayed by flow cytometry. The flow cytometry assay and RNA silencing of CD24 on Huh-7 and BEL-7402 cells was performed as described previously [12].

### Antibody-dependent cellular cytotoxicity (ADCC)

The CytoTox 96 Nonradioactive Cytotoxicity assay (Promega, Madison, USA) was used for the cytotoxicity assay. Huh-7/BEL-7402 cells were co-cultured with various numbers of PBMCs or NK-92 cells in the presence or absence of treatment for 4 h at 37°C. LDH analysis was performed according to the manufacturer's protocol [12]. The controls were set as groups of spontaneous LDH release in effector and target cells and target maximum release. The calculation of cytotoxicity percentage was determined as: %Cytotoxicity = [(experimental - effector spontaneous - target spontaneous)/(target maximum - target spontaneous)]×100.

### Dynamics and targeting capability by near infrared (NIR) imaging *in vivo*

The near infrared dye IRB-NHS (10 mg/ml) (Shanghai Sciencelight Biology Science & Technology Co., Ltd., Shanghai, China) in 20 μl of dimethylsulfoxide (DMSO) was added to 4 ml of hG7-BM3 (10 mg/ml) in phosphate buffered saline (PBS, 0.01 M, pH 7.4). After a 2 h reaction at 25°C, the product was applied to a Sephadex G-75 molecular sieve column to remove free IRB-NHS, and the purified probes were collected and named IRB-hG7-BM3. A total of 10 Huh-7-bearing mice were divided into two groups. IRB-hG7-BM3 (250 nmol/kg) was intravenously injected into the mice. In addition, the free hG7-BM3 (12.5 μmol/kg) was mixed with IRB-hG7-BM3 (250 nmol/kg) to evaluate competitive blocking. Then, a near-infrared fluorescence imaging system conducted the fluorescence imaging at serial time points. The analysis of the region of interest (ROI) function was utilized for analyzing tumor/normal tissue ratios (T/N ratio) [11].

### Antibody internalization assay

To assess the hG7-BM3 internalization *in vitro*, flow cytometry and confocal microscopy analyses were used in different cell lines. First, 5×10^5^ tumor cells (Huh-7/BEL-7402) were rinsed twice, split into eight experimental groups and incubated with 200 nM hG7-BM3 at 37°C. Timed groups (0, 2, 5, 15, 30, 40, 60, 90 min) were placed on ice and internalization was stopped. The flow cytometry assay followed the steps described above. The internalization percentage was calculated from the mean fluorescence intensities (MFI) as: % Internalization = [(MFI _TimeX_-MFI_background_) / (MFI_Time0_ - MFI_background_)] ×100.

We also used a fluorescence microscope to observe the internalization effect directly. hG7-BM3 was labeled with the visible fluorescent dye RhodamineB (Beyotime Institute of Biotechnology, Shanghai, China). The coupling method followed that described previously by Li Ding and the probes were named RhB-hG7-BM3. First, 5×10^4^ Huh-7/BEL-7402 cells were cultured overnight at 37°C and then incubated with 1 μM of RhB-hG7-BM3 for 2 h. After washing with PBS, the cells were imaged by a laser confocal microscope (Olympus FV1100). In addition, the free hG7-BM3 (50 μM) was mixed with RhB-hG7-BM3 (1 μM) to evaluate the competitive blocking [47].

### Preparation of ADC (hG7-BM3-VcMMAE)

hG7-BM3 was partially reduced with 2.5 mole equivalent of tris (2-carboxyethyl) phosphine hydrochloride (TCEP·HCl, Thermo scientific, USA) for 1 h. The buffer was exchanged with PBS containing 1 mM diethylenetriamine pentaacetic acid (DTPA) by centrifugal ultrafiltration (50 kDa, Millipore) after reduction. Then, the conjugation reaction mixture was prepared by gently dropping VcMMAE (DC Chemicals, Shanghai, China) dissolved in dimethyl-sulfoxide (DMSO) into the reduced hG7-BM3 for 30 min, and the VcMMAE / hG7-BM3 ratio was determined to be 4:1. All reactions were carried out at 4°C. Excess VcMMAE and TCEP were removed by centrifugal ultrafiltration. The characterization of hG7-BM3-VcMMAE was confirmed with 8% SDS-PAGE.

### Hydrophobic interaction chromatography (HIC) analysis

ADC samples were analyzed by HIC using an Agilent 1200 HPLC system (Wilmington, DE, USA) with a nonporous TSKgel Butyl-NPR column with a 2.5 μm particle size in a dimension of 4.6 mm×35 mm (Tosoh Bioscience, Tokyo, Japan). Mobile phase A was an aqueous solution of 1.5 M ammonium sulfate and 25 mM sodium phosphate at pH 7.0; mobile phase B was a 75% (v/v) aqueous solution of 25 mM sodium phosphate at pH 7.0 and 25% (v/v) isopropyl alcohol (IPA). The gradient was 10% B to 100% B over 20 min at a 0.5 mL/min flow rate, and the UV detection wavelength was 280 nm. The weighted average drug/antibody ratio was determined by peak area integration, DAR=Σ(Weighted Peak Area)/100, as previously reported by Jun Ouyang [48].

### Anti-tumor efficacy in xenograft tumor models

HCC-bearing mice were randomly assigned to six groups (6 per group) to receive treatments with hG7-BM3-VcMMAE (2 mg/kg, 0.5 mg/kg), hG7-BM3 (2 mg/kg), MMAE (Selleckchem, Houston, USA) (0.02 mg/kg), a combination (2 mg/kg hG7-BM3 and 0.02 mg/kg MMAE) or saline as the vehicle control intravenously administered once every three days for 3 weeks. The body weights and tumor volumes were measured every other day. Tumor volumes were calculated using the formula V=LW^2^/2 (L: longer diameter of tumor, W: shorter diameter of vertical direction). The mice were sacrificed 3 weeks after treatment and tumors were prepared for immunohistochemistry (IHC) analysis. Paraffin sections were cut into 5 μm sections and fixed in 4% paraformaldehyde. For immunohistochemical (IHC) staining, the sections were incubated with anti-Ki67, anti-cleaved caspase-3 antibody (Cell Signaling Technology, Boston, USA), and horseradish peroxidase-labeled secondary antibody and analyzed with the Vectastain ABC Kit (Dako, Copenhagen, Denmark). Tumor sections were viewed and photographed using the Zeiss Axio Vert A1 microscope (Carl Zeiss, Thornwood, NY, USA) [12].

### Statistical analysis

The data are indicated as means ± standard deviation (SD). Significance levels were estimated using the student's t test and P values of 0.05 or less were considered statistically significant. The calculation was performed with the GraphPad Prism software (San Diego, CA).

## SUPPLEMENTARY MATERIALS FIGURES



## Abbreviations

CD24: cluster of differentiation 24
HCC: hepatocellular carcinoma
CDR: complementarity determining region
VL: light chain variable region
VH: heavy chain variable region
3-D: three dimensional
ADCC: antibody-dependent cellular cytotoxicity
ADC: antibody-drug conjugate
MOE: molecular operating environment
T-IC: tumor-initiating cell
HAMA: human anti-mouse antibody
FR: framework regions
PDB: Protein Data Bank
PBMCs: peripheral blood mononuclear cells
LDH: lactate dehydrogenase
NIR: near infrared
DMSO: dimethylsulfoxide
MFI: mean fluorescence intensities
HIC: hydrophobic interaction chromatography
HPLC: high performance liquid chromatography
IHC: immunohistochemistry
RMSD: Root Mean Square Deviation
GPI: glycosyl phosphatidyl inositol
MMAE: Monomethyl auristatin E
