# Supplementary material for: Engineering a high-affinity humanized anti-CD24 antibody to target hepatocellular carcinoma by a novel CDR grafting design
Source: Oncotarget. 2017 Apr 19;8(31):51238–52. doi: 10.18632/oncotarget.17228 (PMC5584245; doi:10.18632/oncotarget.17228)
Supplement: Supplementary file 1 [file oncotarget-08-51238-s001.pdf]

Engineering a high-affinity humanized anti-CD24 antibody to target hepatocellular carcinoma by a novel CDR grafting design

SUPPLEMENTARY MATERIALS

SUPPLEMENTARY METHODS AND RESULTS

Stability in human serum

The cG7 and hG7s was diluted to 200nM in freshly isolated human serum and incubated at 37°C and 5% CO_2 for up to 7 days. The cG7 or hG7s binding activity to Huh-7 cells was determined at different time points (0, 1, 3, 5, and 7 day for the incubation in human serum) by flow cytometry. We measured the binding ratio of the cG7 or hG7s at each time point and calculated the percentage of activity (%activity = binding ratio of the sample treated with human serum/ binding ratio of the sample at 0 day). As shown in Supplementary Figure 2, we measured the binding ratio of the cG7 or hG7s at each time point and calculated the percentage of activity. hG7-BM1, hG7-BM3 and cG7 retained 49.4%, 66.5% and 42.4% of their binding activity when incubated for 7 days at 37°C in human serum, respectively. cG7 and hG7-BM1 lost about 50% of its activity after just 1 day. Whereas hG7-BM3 retained more than 60% of its activity after 7 days, indicating that hG7-BM3 is a highly stable humanized antibody.

The immunogenicity assay of humanized antibody

Peripheral blood mononuclear cells (PBMCs) were isolated from healthy donors' PBL by Ficoll–Hypaque density gradient centrifugation (Lympholyte-H kit, Burlington, Canada). Serum-free AIM-V medium was used to culture PBMCs and 1% beta-mercaptoethanol was added for 2 hours. 1000 IU/ml IL-4 and GM-CSF (Peprotech, London, UK) were added into medium for 5 days. Then 0.2 IU/ml TNF-α and 50 IU/ml IL-1α were supplemented and cells were cultured for 2 days. On day 7, 50 mg/ml mitomycin C (MMC) was used to stop the differentiation of dendritic cells (DC). The mature DC were harvested by centrifuging at 600 g and 1×10^4 cells were added in 96-well U-bottomed plates. The $CD4^+$ cells were enriched using anti-CD4 mAb-coated immunomagnetic beads and labeled with carboxyfluorescein succinimidyl ester (CFSE). 1×10^5 $CD4^+$cells were co-cultured with DC in 96-well plates. Chimeric antibodies and humanized antibodies were added at 5mM to 96-well plates. T cell proliferation was determined based on the CFSE dilution assay by flow cytometry and the results were converted to a Stimulation Index(SI). SI equal to or greater than 1.9 (SI≥1.90) are deemed positive. SI = percentage of proliferative cells (antibody group) / percentage of proliferative cells (PBS group).

After incubation of matured DC cells, humanized antibody hG7-BM3; recombinant human antibody mAb04; chimeric antibodies cG7 and Cetuximab were preloaded with autologous $CD4^+$ T cells for seven days. Based on CFSE dilution method, $CD4^+$ T cells loaded with hG7-BM3 showed lower proliferation, when compared to those loaded with chimeric antibodies. As shown in Supplementary Figure 3 the T-cell Stimulation Index(SI) of the hG7-BM3 was less than 1.9, that result was not deemed positive. What's more, hG7-BM3 and mAb04 had similar SI and less than chimeric antibodies cG7 and Cetuximab, indicating that the immunogenicity of hG7-BM3 was lower than that of parental antibody.

The body weights analysis

It's necessary to monitor the mice body weight because it's an important index for drug toxicity. The body weights were measured every other day.

The body weights of mice in hG7-BM3-VcMMAE and hG7-BM3 groups gradually increased within the treatment period, indicating no apparent physical toxicity, And there was weight loss observed the group treated with MMAE, indicating that MMAE has toxic or side effects to mice.

SUPPLEMENTARY FIGURES

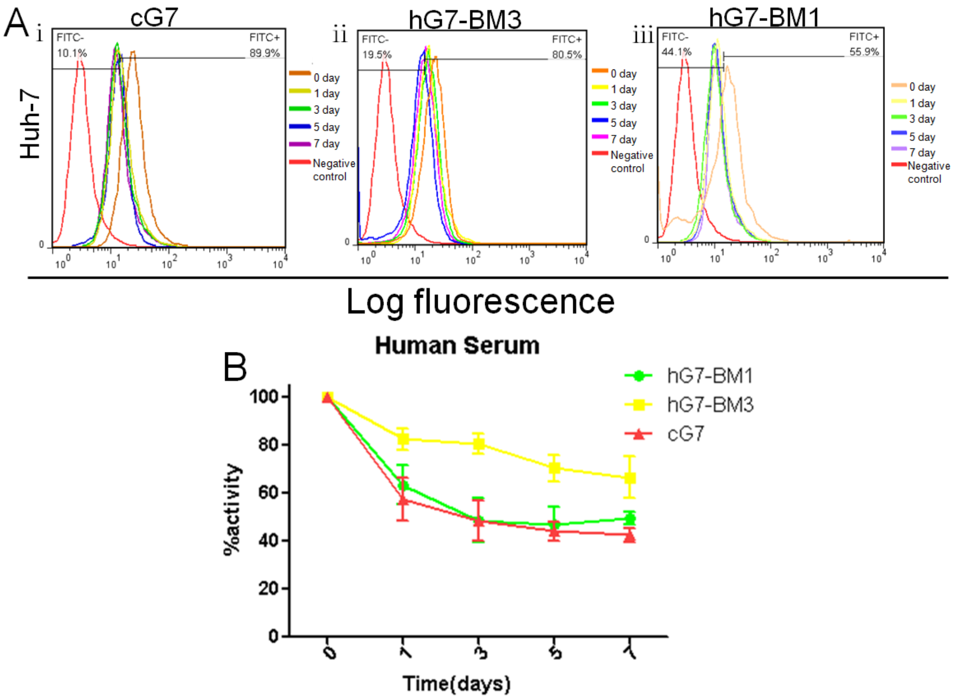

Supplementary Figure 1: hG7-BM3 showed a higher stability in human serum. The hG7-BM3 were incubated at and 37°C for various time periods (up to 7 days) in human serum. **(A)** The binding ratio of the cG7 or hG7s at each time point was determined by flow cytometry. **(B)** The percentage of activity curves of each group (%activity = binding ratio of the sample treated with human serum/ binding ratio of the sample at 0 day).

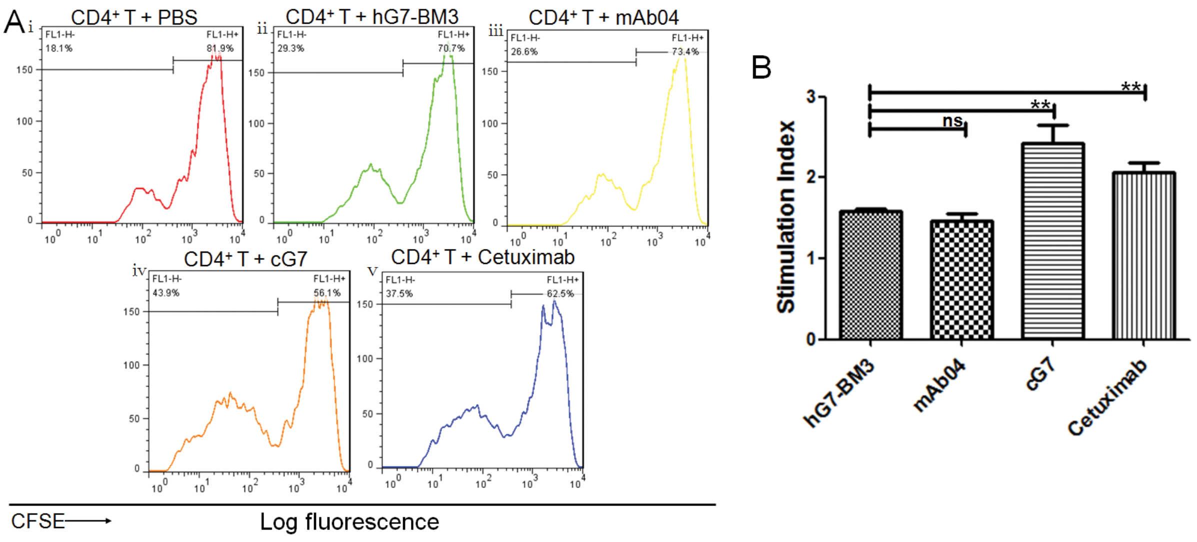

Supplementary Figure 2: hG7-BM3 showed a lower induction of CD4+ T cell proliferation. **(A)** CD4+ T cells were previously labeled with CFSE and the progeny, the percentage of T cells that proliferated, was estimated by flow cytometry, based on the CFSE dilution. The histograms show representative experiments where T cells were co-cultured with: hG7-BM3, mAb04, cG7, Cetuximab. Unstimulated T cells (Ai) served as negative control. **(B)** Histogram represent the T-cell Stimulation Index (SI) of different antibodies. Data were given as the mean ± SD (n = 3). $^{**}p < 0.01$, ns: no significance.

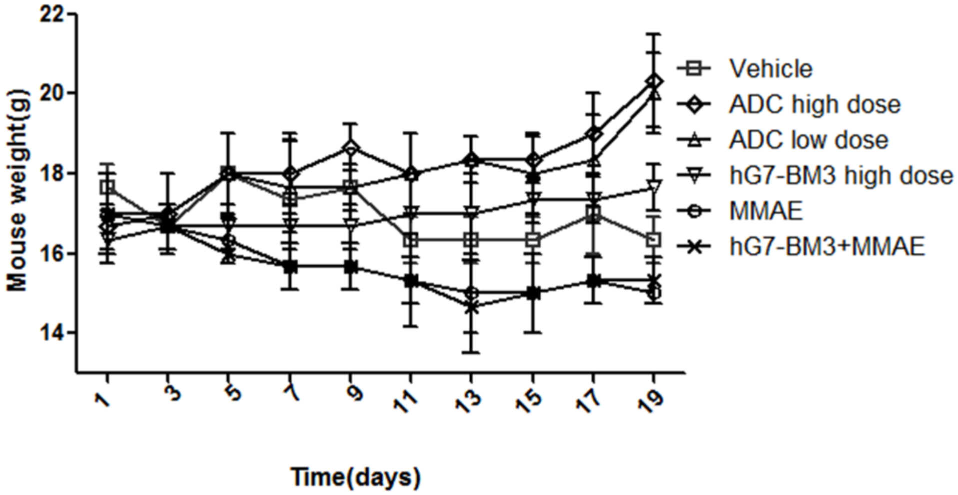

Supplementary Figure 3: Body weights of mice in different groups. There was no weight loss observed in both groups treated with hG7-BM3-VcMMAE, indicating that the treatments were well tolerated, but a decrease of body weight in the MMAE-treated group was observed after 19 days treatment.
